# Time-averaged albumin predicts the long-term prognosis of IgA nephropathy patients who achieved remission

**DOI:** 10.1186/1479-5876-12-194

**Published:** 2014-07-10

**Authors:** Zhaohui Ni, Yanhong Yuan, Qin Wang, Liou Cao, Xiajing Che, Minfang Zhang, Yuanyuan Xie, Chaojun Qi, Shan Mou

**Affiliations:** 1 Department of Nephrology, Molecular Cell Lab for Kidney Disease, Ren Ji Hospital, School of Medicine, Shanghai Jiao Tong University, 1630 Dongfang Road, Shanghai 200127, China

**Keywords:** IgA nephropathy, Remission, Renal progression, Risk factor, Time-averaged serum albumin

## Abstract

**Background:**

Primary IgA nephropathy (IgAN) is the most common form of idiopathic glomerulonephritis worldwide. Although most patients are able to achieve remission with the current therapy, a large number of patients will still progress to end-stage renal disease. This study aimed to evaluate kidney disease progression and the risk factors for progression in IgAN patients who achieved remission.

**Methods:**

Patients from a prospective database with IgAN were included in this study. All the subjects had achieved a complete remission (CR) or partial remission (PR) following 6 months of therapy. Renal survival and the relationship between the clinical parameters and composite renal outcomes were assessed.

**Results:**

The study comprised 878 IgAN patients recruited between January 2005 and December 2010. Overall, 632 patients were enrolled in this study. The data from the 369 patients who achieved remission were analyzed; the mean follow-up time was 49 months. The median serum creatinine (SCr) concentration at baseline was 91.3 μmol/L, and the time-averaged creatinine (TA-SCr) was 91.8 μmol/L. The mean serum albumin (ALB) level at baseline was 39.4 g/L, and the time-averaged serum albumin (TA-ALB) was 42.1 g/L. Multivariate Cox regression analyses revealed that the TA-ALB and TA-SCr levels were independently associated with the composite renal outcome. The patients with a TA-SCr value > 120 μmol/L and a TA-ALB level < 38 g/L were less likely to recover from renal progression.

**Conclusion:**

The strong predictive relationship of low TA-ALB and high TA-SCr levels with progression observed in this study suggests that TA-ALB may serve as a marker of the long-term renal prognosis of IgAN patients who have achieved remission.

## Introduction

Primary IgA nephropathy (IgAN) is a very common idiopathic glomerulonephritis (GN) throughout the world [1], especially in China, where IgAN accounts for 58.2% of the GN cases [2]. Studies have confirmed that 1%-2% of IgAN patients will develop end stage renal disease (ESRD) within 1 year of diagnosis [3,4], and approximately 40% of patients will ultimately develop ESRD within 20 years [5,6]. A wide variety of treatments have been attempted to slow the progression of IgAN. Based on the evidence, most treatment guidelines for IgAN recommend optimal blood pressure control and suggest adding steroids for those patients with persistent proteinuria, regardless of supportive therapy [7]. However, few studies have evaluated the progression of and risk factors for the progression of IgAN under the current treatment regimens.

Several clinical researchers [8-10] have confirmed the prognostic value of certain clinical and biochemical parameters for the outcomes of patients with IgAN. Systolic hypertension, massive proteinuria, renal impairment, serum albumin (ALB) level [11,12], and severe histological findings on a renal biopsy have been reported as risk factors for ESRD [13]. Other risk factors regarding the clinical course [5,14-18] are numerous and controversial in the literature, including age at disease onset, gender, obesity, hemoglobin levels, hypertriglyceridemia, inappropriate lifestyles, geography, and various immunogenetic markers. One of the problems in the field is that previous studies have consistently identified various clinicopathologic parameters at the time of diagnosis, but the patients were not followed long-term in most cases. By analyzing a large number of patients, this study aimed to clarify the long-term renal survival and related risk factors for progression in IgAN patients who achieved remission with the current therapy.

## Materials and methods

### Subjects

The study comprised 878 IgAN patients recruited between January 2005 and December 2010 from the Department of Nephrology, Ren Ji Hospital, Shanghai, China. All the patients had definitive pathological data with predominant (at least 1+) mesangial staining for IgA according to immunofluorescence staining and electron-dense deposits within the mesangium according to electron microscopy analysis. Patients who presented at younger than 18 years of age (n = 30), were pregnant (n = 3), had acute kidney failure (n = 12), had less than 36 months of follow-up (n = 160), had systemic inflammation such as Henoch–Schönlein purpura (n = 6), or had chronic advanced liver disease (n = 35) were excluded. A total of 632 patients were included. The study was approved by the Ethics Committee of Ren Ji Hospital, and all the participants gave written informed consent. All the kidney biopsy slides were reviewed by an experienced renal pathologist.

Only those subjects in whom the treatment response evaluated at 6 months after the initiation of therapy met the criteria for a complete remission (CR) or partial remission (PR) were included in the present study. A CR was defined as the absence of proteinuria, the normalization of all biochemical findings, and a lack of worsening of renal function. A PR was defined as a > 50% reduction in proteinuria from baseline. No response (NR) was defined as a < 50% reduction in proteinuria or an increase in proteinuria, with or without renal deterioration. In the end, 369 patients who met the remission criteria were enrolled.

**Figure 1:**
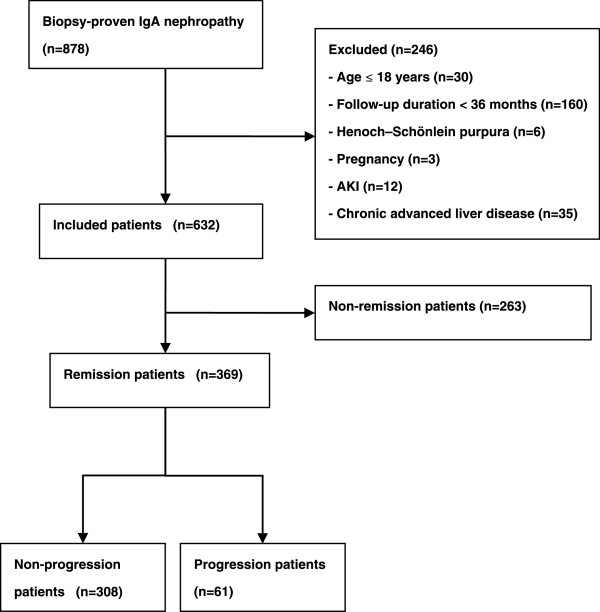
**A flow diagram of the study.** AKI: Acute kidney injury.

At the end of the follow-up, the estimated glomerular filtration rate (eGFR) values were evaluated. The primary outcome was a doubling of the baseline serum creatinine (SCr); secondary outcomes included ESRD and death. Patients were classified with progression when their eGFR values decreased (below the normal range) ≥ 5 ml/min/1.73 m^2^/year or reached the endpoint during the follow-up period. The patients who stabilized or had a slower decrease in the eGFR were considered as non-progression patients. Among the 369 remission patients, 61 were progression patients, and 308 subjects were non-progression patients (Figure 1).

### Clinical, biochemical and histopathological data collection

The patients had regular follow-up visits at intervals of at least 3–6 months. All the data were collected prospectively. The baseline clinical and demographic data were recorded at the time of the renal biopsy. The demographic data included age and gender. The clinical parameters included systolic blood pressure (SBP), diastolic blood pressure (DBP), blood erythrocytes (RBC), blood leukocytes (WBC), thrombocytes (PLT), hemoglobin (Hb), SCr, serum uric acid (UA), blood urea nitrogen (BUN), ALB, glutamic-pyruvic transaminase (ALT), glutamic-oxaloacetic transaminase (AST), triglyceride levels (TG), serum total cholesterol levels (TC), high-density lipoprotein (HDL), low-density lipoprotein (LDL), the erythrocyte sedimentation rate (ESR), complement 3 (C3) levels, serum immunoglobulin (IgA, IgG, IgM) levels, 24-h urinary protein excretion (UPE), hematuria (uRBC/HP), and the eGFR at the diagnosis of IgAN and during the follow-up.

The eGFR was calculated using the Modification of Diet in Renal Disease (MDRD) study equation: eGFR (ml/min/1.73 m^2^) = 1.8 × [SCr (mg/dl)]^-1.154^ × (age)^-0.203^ × (0.742 if female) [19]. Chronic kidney disease (CKD) was classified based on the Kidney Disease Outcomes Quality Initiative (K/DOQI) practice guidelines.

For each patient, proteinuria was measured via the UPE. An average UPE was determined for each 3-month block during the follow-up; the average of the UPE values for every 3-month period was represented by the time-averaged UPE (TA-UPE) [20]. The time-averaged serum albumin (TA-ALB), time-averaged serum creatinine (TA-SCr), time-averaged blood urea nitrogen (TA-BUN), and time-averaged eGFR (TA-eGFR) values were calculated using the same method as that used for the TA-UPE.

Most of the patients were treated according to the accepted standards at our center as follows: (1) full doses of ACEis (angiotensin converting enzyme inhibitors) and/or ARBs (angiotensin receptor blockers) were recommended for all the patients with proteinuria or hypertension; (2) steroids were used in cases of massive proteinuria (>1 g/d); and (3) other immunosuppressive agents were considered in patients with impaired kidney function or rapidly progressing kidney function decline (an increase in SCr > 15% in the year before entry into the trial) [21]. Renal lesions were graded according to the Lee’s classification at the time the database was established [22].

### Statistical analyses

Analyses of the data were carried out using SPSS software (version 13: SPSS, Chicago, IL). For comparison of the continuous variables between the groups, Student’s *t*-test was used if the variables had a normal distribution, and a Mann–Whitney *U*-test was used if there was not a normal distribution. The chi squared test was employed for the categorical variables. Cox’s proportional hazards models for estimating the hazard ratios and the 95% confidence intervals (CI) were used to identify the predictive factors for the development of IgAN progression. The multivariate models used a stepwise forward selection procedure based on a likelihood-ratio test with P > 0.10 for the removal and P < 0.05 for the entry of the variables. Receiver operating characteristic curves (ROC) were established to determine the optimal cutoff value of a risk factor for predicting renal progression. In addition, ROC curves were also analyzed when two risk factors were combined. Renal survival was estimated with the Kaplan–Meier method, and comparisons were performed using the log-rank test. The results were reported as the means ± standard deviation (SD). Values of P less than 0.05 were considered statistically significant.

## Results

### Baseline demographic and clinical data

From 2005 to 2010, 878 patients with primary IgAN were recorded in the Registry Database, among whom 246 cases were excluded and 632 cases were included in this study. The data from a total of 369 patients with IgAN who achieved remission 6 months following therapy were utilized in the analyses. The clinical characteristics at baseline and during the follow-up were summarized in Tables 1 and 2. All the subjects were Chinese. There were 184 males and 185 females in the study, and the mean age at biopsy was 37.2 ± 12.3 years. The mean SBP and DBP at baseline were 123.8 ± 15.9 mm Hg and 79.3 ± 9.5 mm Hg, respectively. The mean eGFR was 83.6 ± 28.4 ml/min/1.73 m^2^. The median and interquartile range (IQR) values for the initial proteinuria were 2.1 and 1.2-3.2 g/d, respectively. Moreover, 17% of the patients had ALB levels less than 35 g/L, and the mean ALB level was 39.4 ± 5.5 g/L. The patients were followed for 49.0 (38.0-65.8 months) months, and 61 of these patients reached the endpoint of the follow-up.

**Table 1:** Baseline clinical characteristics and renal parameters of patients with IgAN

**Characteristics**	**Remission patients**	**Non-progression patients**	**Progression patients**	**P-value**
	**(n = 369)**	**(n = 308)**	**(n = 61)**	
Age (years)	37.2 ± 12.3	37.0 ± 1.7	38.1 ± 3.9	NS
Gender: Female n (%)	185 (50.1)	160 (51.9)	25 (40.9)	NS
SBP (mm Hg)	123.8 ± 15.9	123.5 ± 2.3	125.0 ± 3.7	NS
DBP (mm Hg)	79.3 ± 9.5	79.2 ± 1.4	79.8 ± 2.8	NS
RBC (X 10^12^/L)	4.4 ± 0.5	4.4 ± 0.2	4.5 ± 0.3	NS
WBC (X 10^9^/L)	7.2 ± 1.7	7.0 ± 0.3	7.8 ± 0.4	NS
Hb g/L	133.7 ± 18.8	134.3 ± 2.9	131.3 ± 5.4	NS
PLT (X 10^9^/L)	209.7 ± 63.8	215.5 ± 10.8	191.6 ± 23.6	NS
ALB g/L	39.4 ± 5.5	39.9 ± 0.8	37.7 ± 1.5	NS
ALT IU/L	16.0 (12.0-20.5)	16.0 (13.0-22.0)	15.0 (11.0-19.0)	NS
AST U/L	19.7 ± 8.5	20.4 ± 1.7	17.5 ± 0.9	NS
TG mmol/L	1.6 (1.1-2.3)	1.56 (1.1-2.4)	1.9 (1.3-2.2)	NS
TC mmol/L	5.0 (4.3-6.0)	4.97 (4.3-6.0)	5.1 (4.5-5.9)	NS
HDL mmol/L	1.29 ± 0.5	1.3 ± 0.1	1.2 ± 0.1	NS
LDL mmol/L	3.35 ± 1.7	3.4 ± 0.3	3.1 ± 0.2	NS
FBG mmol/L	4.9 ± 0.5	4.93 ± 0.1	4.8 ± 0.2	NS
SCr μmol/L	91.3 ± 36.4	86.2 ± 4.7	112.8 ± 11.9	0.02
BUN mmol/L	6.1 ± 2.4	5.8 ± 0.3	7.7 ± 0.9	0.03
eGFR (ml/min/1.73 m^2^)	83.6 ± 28.4	87.7 ± 3.9	66.7 ± 7.8	0.03
UA μmol/L	337.0 (280.9-426.5)	327.5 (277.4-414.0)	370.0 (326.5-514.5)	NS
Ca mmol/L	2.3 ± 0.1	2.3 ± 0.02	2.3 ± 0.1	NS
P mmol/L	1.2 ± 0.2	1.23 ± 0.05	1.2 ± 0.04	NS
ESR mm/h	22.0 (10.0-34.0)	22.0 (9.0-34.0)	24.0 (11.0-33.0)	NS
IgA g/L	2.9 ± 0.9	2.8 ± 0.2	3.1 ± 0.2	NS
IgG g/L	12.3 ± 3.2	12.7 ± 0.6	11.4 ± 0.9	NS
IgM g/L	1.3 ± 0.8	1.4 ± 0.2	1.1 ± 0.2	NS
C3 g/L	1.05 ± 0.2	1.06 ± 0.04	1.0 ± 0.1	NS
UPE (g/d)	2.1 (1.2-3.2)	1.8 (1.2-3.1)	2.4 (2.0-3.6)	NS
uRBC /HP	28.4 (7.5-50.0)	29.1 (7.2-50.0)	14.0 (9.4-29.1)	NS
CKD stage n (%)				
Stage 1	310 (84.0)	264 (85.7)	46 (75.4)	
Stage 2	37 (10.0)	29 (9.4)	8 (13.1)	
Stage 3	22 (6.0)	15 (4.9)	7 (11.5)	
Stage 4	0	0	0	
Stage 5	0	0	0	NS
Renal biopsy Lee’s classification n (%)				
Grade I-II	13 (3.5)	13 (4.2)	0	
Grade III	194 (52.6)	163 (52.9)	31 (50.8)	
Grade IV	131 (35.5)	107 (34.7)	24 (39.3)	
Grade V	31 (8.4)	25 (8.2)	6 (9.9)	NS

Values are given as the means ± standard deviation (SD) if the variables showed a normal distribution and as medians (IQR) if the variables did not show a normal distribution; n (%) was used for the categorical variables.

SBP: systolic blood pressure; DBP: diastolic blood pressure; RBC: blood erythrocytes; WBC: blood leukocytes; Hb: hemoglobin; PLT: thrombocytes; ALB: serum albumin; ALT: glutamic-pyruvic transaminase; AST: glutamic-oxaloacetic transaminase; TG: triglyceride levels; TC: Serum total cholesterol levels; HDL: high density lipoprotein; LDL: low density lipoprotein; FBG: fasting blood glucose; SCr: serum creatinine; BUN: blood urea nitrogen; eGFR: estimated glomerular filtration rate; UA: serum uric acid; Ca: serum calcium levels; P: serum inorganic phosphorus levels; ESR: erythrocyte sedimentation rate; IgA, IgG, IgM: serum immunoglobulin levels; C3: complement 3 levels; UPE: 24-h urinary protein excretion; uRBC/HP: hematuria.

**Table 2:** Comparison of characteristics between progression and non-progression patients during the follow-up

**Characteristics**	**Remission patients**	**Non-progression patients**	**Progression patients**	**P-value**
	**(n = 369)**	**(n = 308)**	**(n = 61)**	
ALB g/L at month 3	40.9 ± 4.9	41.3 ± 0.6	39.0 ± 1.7	NS
ALB g/L at month 6	42.9 ± 4.2	43.5 ± 0.6	40.5 ± 1.3	0.02
TA-ALB g/L	42.1 ± 3.8	42.7 ± 0.5	39.4 ± 1.2	0.01
SCr μmol/L at month 3	90.2 ± 35.5	82.7 ± 3.9	121.6 ± 13.9	0.01
SCr μmol/L at month 6	88.2 ± 37.5	80.3 ± 4.2	121.3 ± 14.1	0.002
TA-SCr μmol/L	91.8 ± 46.8	80.5 ± 4.2	136.7 ± 21.0	0.002
BUN mmol/L at month 3	7.4 ± 2.9	6.8 ± 0.3	9.8 ± 1.1	0.01
BUN mmol/L at month 6	6.5 ± 2.3	6.1 ± 0.3	8.0 ± 0.9	0.02
TA-BUN mmol/L	6.6 ± 2.2	6.1 ± 0.2	8.6 ± 0.9	0.004
eGFR (ml/min/1.73 m^2^) at month 3	85.5 ± 33.1	90.8 ± 4.4	63.4 ± 8.2	0.01
eGFR (ml/min/1.73 m^2^) at month 6	88.9 ± 33.7	95.1 ± 4.6	63.1 ± 7.8	0.003
TA-eGFR (ml/min/1.73 m^2^)	88.5 ± 33.2	95.1 ± 4.5	61.9 ± 8.1	0.002
UPE (g/d) at month 3	0.7 (0.4-1.3)	0.6 (0.2-1.0)	1.3 (0.7-2.0)	0.04
UPE (g/d) at month 6	0.4 (0.2-0.7)	0.4 (0.1-0.7)	0.4 (0.2-1.2)	NS
TA-UPE (g/d)	1.1 (0.6-1.6)	1.0 (0.6-1.4)	1.3 (1.0-2.1)	NS

Values are given as means ± standard deviation (SD) if the variables had a normal distribution and as medians (IQR) if the variables did not have a normal distribution; n (%) was used for the categorical variables.

TA-ALB: time-averaged serum albumin; TA-SCr: time-averaged serum creatinine; TA-BUN: time-averaged blood urea nitrogen; TA-eGFR: time-averaged eGFR; TA-UPE: time-averaged 24-h urinary protein excretion.

### Comparison of the baseline characteristics of the progression and non-progression groups

We compared the baseline characteristics of the progression and non-progression groups (Table 1). Our data revealed that the patients with renal progression had a worse baseline renal function, as represented by the eGFR (66.7 ± 7.8 ml/min/1.73 m^2^ versus 87.7 ± 3.9 ml/min/1.73 m^2^; P = 0.03), the SCr level (112.8 ± 11.9 μmol/L versus 86.2 ± 4.7 μmol/L; P = 0.02), and the BUN (7.7 ± 0.9 mmol/L versus 5.8 ± 0.3 mmol/L; P = 0.03). There were no significant differences in the other baseline characteristics, including gender, age, blood pressure, ALB levels, and so on. The classifications of the renal histopathology were summarized in Table 1, and no significant differences were seen between the progression and non-progression groups.

### Comparison of the characteristics of the progression and non-progression groups during the follow-up

The clinical profiles present during the follow-up after diagnosis were shown in Table 2. The renal function, as represented by the eGFR, SCr and BUN values, was significantly different between the two groups, similar to what was observed at baseline. The level of ALB at 6 months was lower (40.5 ± 1.3 versus 43.5 ± 0.6 g/L; P = 0.02) in patients in the renal progression group than those in the non-progression group. The median value of the UPE at 3 months after the initial biopsy was higher [1.3 (0.7-2.0) g/d versus 0.6 (0.2-1.0) g/d; P = 0.04] in the progression group than in the non-progression group. No significant difference was found in the TA-UPE between the two groups.

The time-averaged characteristics of the two groups were also summarized in Table 2, which indicated that the progressing patients had a lower level of TA-ALB (39.4 ± 1.2 g/L versus 42.7 ± 0.5 g/L; P = 0.01), a lower level of TA-eGFR (61.9 ± 8.1 ml/min/1.73 m^2^ versus 95.1 ± 4.5 ml/min/1.73 m^2^; P = 0.002), a higher level of TA-SCr (136.7 ± 21.0 μmol/L versus 80.5 ± 4.2 μmol/L; P = 0.002), and a higher level of TA-BUN (8.6 ± 0.9 mmol/L versus 6.1 ± 0.2 mmol/L; P = 0.004) than the non-progressing patients.

### Predictors of progression in IgAN patients who achieved remission

Both univariate and multivariate Cox analyses were performed to evaluate the impact of the potential predictors of renal progression. As shown in Table 3, in the univariate analyses, the SCr, BUN, and eGFR values at the baseline and follow-up time periods, the ALB level at the sixth month, and the TA-ALB were statistically significant. Those factors that were significantly correlated with progression on the basis of the univariate analysis were further evaluated with a multivariate analysis. The results revealed that only the TA-ALB and TA-SCr levels were independently associated with renal progression. The adjusted multivariate Cox analysis model indicated that each 1 g/L drop in the TA-ALB level was associated with a 14% increase in the risk of renal progression.

**Table 3:** Factors that were found to affect the long-term prognosis in IgAN patients in the univariate and multivariate COX regression analyses

	**Univariate analysis**				**Multivariate analysis**	
	**HR**	**95% CI**	**P value**	**HR**	**95% CI**	**P value**
ALB g/L at month 6	0.86	0.76-0.96	0.01			NS
TA-ALB g/L	0.8	0.70-0.93	0.002	0.86	0.75-0.98	0.03
Baseline SCr μmol/L	1.02	1.01-1.04	0.003			NS
SCr μmol/L at month 3	1.03	1.02-1.05	<0.001			NS
SCr μmol/L at month 6	1.03	1.02-1.04	<0.001			NS
TA-SCr μmol/L	1.02	1.01-1.03	<0.001	1.02	1.01-1.03	<0.001
Baseline BUN mmol/L	1.37	1.14-1.65	0.001			NS
BUN mmol/L at month 3	1.35	1.15-1.59	<0.001			NS
BUN mmol/L at month 6	1.36	1.10-1.69	0.005			NS
TA-BUN mmol/L	1.56	1.25-1.94	<0.001			NS
Baseline eGFR (ml/min/1.73 m^2^)	0.97	0.94-0.99	0.01			NS
eGFR (ml/min/1.73 m^2^) at month 3	0.96	0.94-0.98	0.004			NS
eGFR (ml/min/1.73 m^2^) at month 6	0.96	0.93-0.98	0.001			NS
TA-eGFR (ml/min/1.73 m^2^)	0.96	0.93-0.98	0.001			NS
UPE (g/d) at month 3	1	1.00-1.002	0.003			NS
TA-UPE (g/d)	1	1.000-1.002	0.01			NS

As mentioned previously, the TA-ALB value was used to quantify the level of ALB during the follow-up of patients with IgAN, and the ROC curves for the TA-ALB were established to determine the optimal cutoff values for predicting a worse outcome (Table 4). The area under the ROC curve (AUC) was 0.82 when the TA-ALB was combined with the ALB value at baseline, at the third month and at the sixth month, indicating that the TA-ALB had a high diagnostic accuracy for an unfavorable renal outcome (sensitivity 90%, specificity 83%).

**Table 4:** Diagnostic performance of ALB for the identification of IgAN progression according to the ROC curve

**Characteristic**	**Area under the curve (AUC)**	**95% CI of AUC**	**P value**	**Cut-off value**	**Sensitivity**	**Specificity**
**One characteristic**						
ALB at month 6	0.72	0.55-0.89	0.02	41.35	0.75	0.74
TA-ALB	0.76	0.59-0.93	0.01	40.89	0.8	0.7
**Two characteristics combined**						
Baseline ALB combined with TA-ALB	0.77	0.62-0.93	0.01		0.8	0.74
ALB at month 3 combined with TA-ALB	0.82	0.65-1.00	0.001		0.9	0.8
ALB at month 6 combined with TA- ALB	0.77	0.60-0.94	0.01		0.8	0.67
**Four characteristics combined**						
Baseline ALB combined with ALB at month 3, ALB at month 6, and TA-ALB	0.83	0.68-0.96	0.001		0.9	0.83

**Figure 2:**
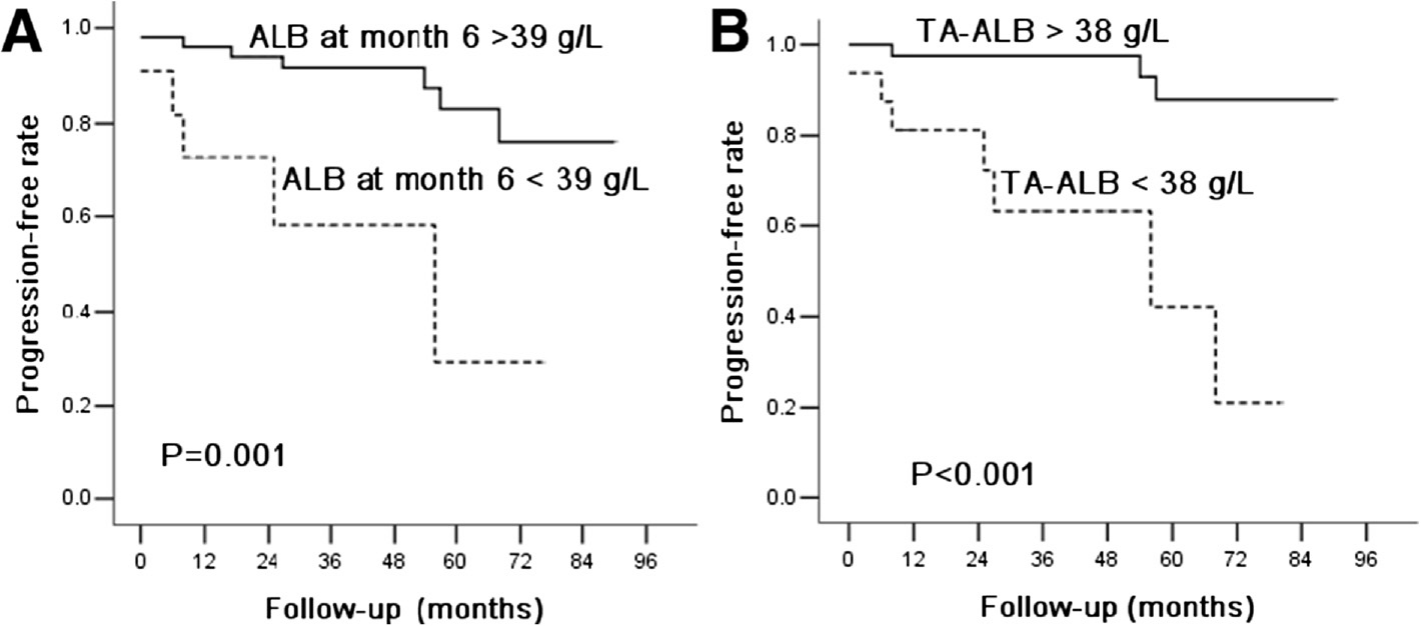
**Kaplan–Meier plots of the renal progression-free rates according to the ALB levels during the long-term follow-up. (A)** ALB levels at month 6, **(B)** TA-ALB levels. Patients with a ALB level at month 6 of < 39 g/L or TA-ALB < 38 g/L had significantly shorter progression-free times (P < 0.05).

**Figure 3:**
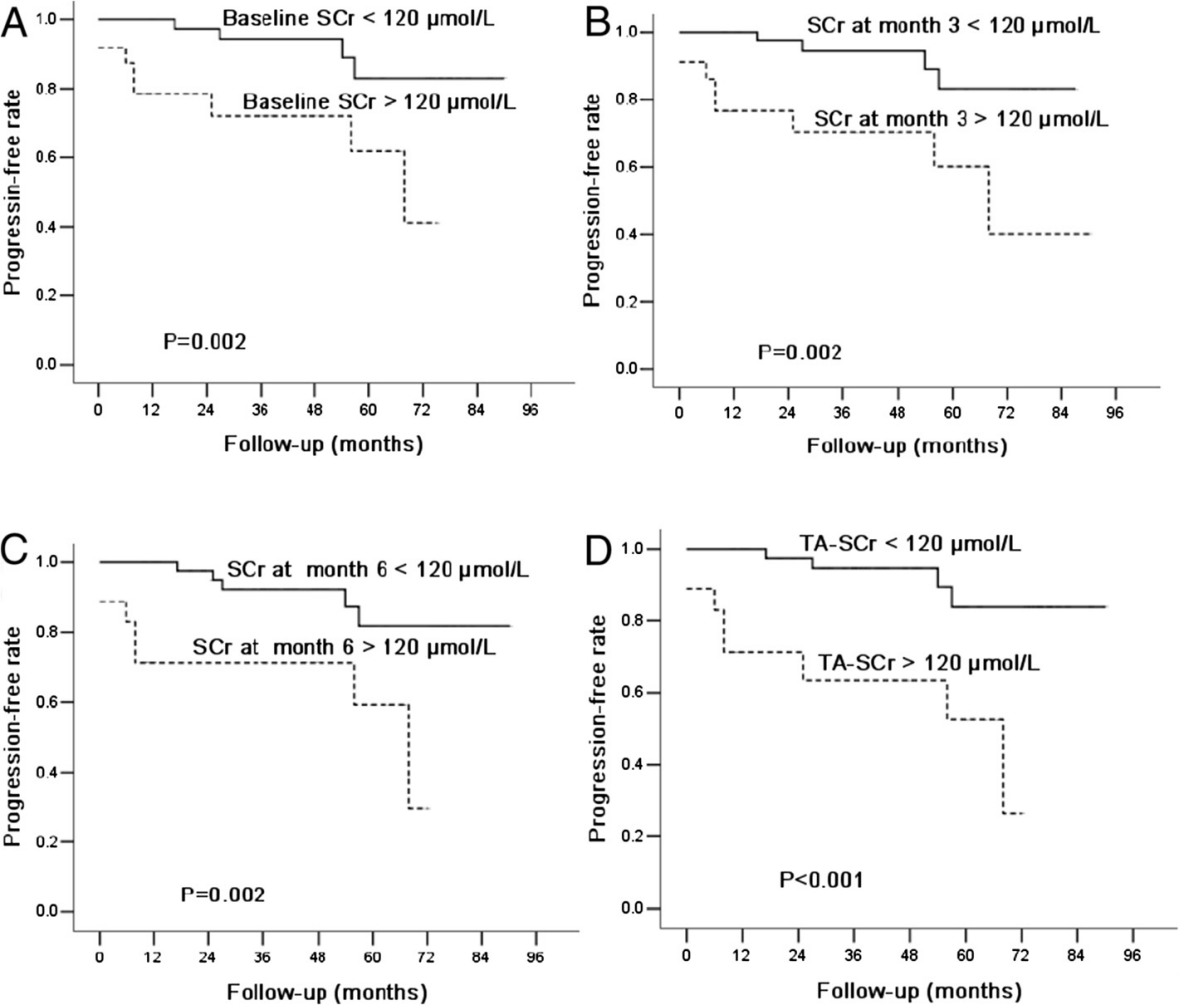
**Kaplan–Meier plots of renal progression-free rates according to the SCr levels at diagnosis and during follow-up. (A)** Baseline SCr levels; **(B)** SCr levels at month 3; **(C)** SCr levels at month 6; **(D)** TA-SCr levels. Patients with baseline SCr > 120 μmol/, SCr > 120 μmol/L at month 3, SCr > 120 μmol/L at month 6, or TA-SCr > 120 μmol/L had significantly reduced progression-free times (P < 0.05).

Based on the Kaplan-Meier analyses, the actual renal survival according to the estimated 36-month risk was plotted in Figures 2 and 3. The optimal cutoff for TA-ALB was 38 g/L, indicating that the relationship between the TA-ALB values and renal outcomes was dramatically altered at levels as low as 38 g/L. Those with SCr levels > 120 μmol/L at baseline, the third month, and the sixth month and ALB levels < 39 g/L at the sixth month had an actual cumulative incidence of reaching the primary endpoint.

## Discussion

IgAN is a progressive disease that is characterized by highly variable clinical courses and outcomes [3,23-25]. Although numerous long-term renal survival analyses of patients with IgAN have been published [4,5,16,26,27], few studies have calculated the long-term renal outcomes of Chinese patients, especially those patients who achieve remission after treatment. Nearly all of the patients received RAS inhibitors. Steroids were considered in patients with UPE > 1 g/d, and other immunosuppressive agents were considered in patients with rapidly progressing renal function decline. Proteinuria declined by approximately 50%, and blood pressure was well controlled among the patients treated with our current therapy regimen. In this large prospective cohort study with more than 360 patients, we evaluated renal progression and the risk factors for renal progression with the current therapy regimen.

Although proteinuria is a known risk factor for the progression of IgAN [13,20,28,29], many important questions regarding the role of proteinuria in the prognosis of IgAN remain. In particular, the timing of the measurements requires clarification. Some studies have proven that UPE at diagnosis is often not a predictor of the outcome according to a Cox regression analysis [25,26]; instead, UPE at 1 year or later may better indicate the prognosis [26]. In our study, we included a cohort of IgAN patients with mild proteinuria (2 g/d); more than 70% of the patients had proteinuria < 1 g/d. After a multivariate Cox analysis, we were unable to provide clear evidence that the baseline UPE was associated with renal outcomes. However, we used the TA-UPE value to represent the average level of proteinuria during the follow-up and demonstrated that, while the TA-UPE was a strong predictor of renal function decline in patients with IgAN according to the univariate analysis, this parameter did not independently contribute to the risk in the multivariate models.

At the time of the renal biopsy, a higher level of SCr constituted an independent risk factor for long-term renal survival. Similar to prior reports [2,4,5,8-10,16,26,27], we confirmed that the baseline SCr and eGFR were associated with renal progression among IgAN patients. We also found that the levels of SCr and the eGFR during follow-up were significantly different between the progression and non-progression patients. The TA-SCr value was an important independent risk factor for renal progression. These clinical parameters are among the most consistently reported predictors of progression, with similar findings observed across multiple cohorts [2,4,5,8-10,16,26,27].

Apart from the traditional risk factors, the ALB level during the follow-up was identified as a novel predictor for the progression of IgAN in this study. The adjusted multivariate Cox analysis model revealed that each 1 g/L drop in the TA-ALB level was associated with a 14% increase in the risk of renal progression. The ROC curve indicated that the combination of the ALB at baseline and at follow-up has a more significant value in the prediction of renal outcome. According to the Kaplan-Meier analysis, we found that the patients with a TA-ALB < 38 g/L had a 10.4-fold increased risk of progression compared with those with a TA-ALB > 38 g/L. These results demonstrate that patients are expected to have more unfavorable outcomes when the ALB level during follow-up is reduced to < 38 g/L. Therefore, we speculate that the optimal goal for TA-ALB among Chinese patients with IgAN should be > 38 g/L.

The ALB level is widely recognized as a biomarker of nutritional status and inflammation [30]. Hypoproteinemia is a common complication of CKD that has recently emerged as an important independent risk factor for kidney disease progression [2,31-36]. To our knowledge, our study is the first to identify ALB during a long-term follow-up as an independent risk factor for renal outcomes in IgAN patients. In a study based on a Chinese population, Chen N et al. [9] demonstrated an independent association of low baseline ALB levels with disease progression among patients with IgAN. Liu ZH et al. [2] found that baseline hypoproteinemia was an independent risk factor for an unfavorable IgAN outcome. In contrast to these studies, we did not find a relationship between baseline ALB and renal progression. Instead, our study provides strong support for the predictive value of ALB during a long-term follow-up for the assessment of renal progression risk. In this study, we used TA-ALB values to represent the average level of ALB during the follow-up and demonstrated that the TA-ALB value was a strong predictor of renal progression in patients with IgAN.

In addition, our findings did not confirm any independent association of blood pressure or pathological grading with renal survival. This finding is partly due to the fact that most patients had a normal arterial pressure level after the administration of anti-hypertensive agents, such as full doses of ACEis and/or ARBs. Furthermore, our study applied Lee’s pathological classifications, which might have a limited predictive capacity compared with the Oxford classifications. The Oxford classification system is based upon four scores (mesangial hypercellularity, segmental glomerulosclerosis, endocapillary hypercellularity, and tubular atrophy/interstitial fibrosis) and constitutes an effective method for predicting renal outcomes [37,38].

This study is unique in that it identified predictors for clinical outcomes among a pool of IgAN patients who achieved either a CR or a PR. In addition, this IgAN study also has a relatively longer follow-up time, involving more than 360 patients followed for 100 months. The strengths of this study include the large number of IgAN patients, a uniform therapy strategy, and the robust database, for which all the data were collected at regular 3-month intervals. However, the results of this study are limited by its location at a single medical center, and most of the patients recruited came from the southern regions of China. Furthermore, the renal function of most of the patients at the beginning of the trial period was good, and approximately 94% of the patients were in CKD stage 1–2. Therefore, the analyses had an inherent selection bias for disease severity and treatment choice. Thus, more studies are required to evaluate the progression of IgAN in patients under the current treatment regimen in the future.

## Conclusion

In summary, our data clearly showed that the TA-SCr and TA-ALB levels were associated with renal outcomes in patients achieving remission under the current therapeutic regimens for IgAN. Furthermore, ALB, especially during the follow-up, is a potential predictor for IgAN prognostic outcomes.

## Abbreviations

ALB: Serum albumin; ALT: Glutamic-pyruvic transaminase; AST: Glutamic-oxaloacetic transaminase; BUN: Blood urea nitrogen; Ca: Serum calcium levels; C3: Complement 3 levels; CR: Complete remission; DBP: Diastolic blood pressure; eGFR: Estimated glomerular filtration rate; ESR: Erythrocyte sedimentation rate; ESRD: End stage renal disease; FBG: Fasting blood glucose; GN: Glomerulonephritis; Hb: Hemoglobin; HDL: High density lipoprotein; IgAN: IgA nephropathy; IgA: IgG, IgM, Serum immunoglobulin levels; LDL: Low density lipoprotein; PR: Partial remission; PLT: Thrombocytes; P: Serum inorganic phosphorus levels; RBC: Blood erythrocytes; SBP: Systolic blood pressure; SCr: Serum creatinine; TG: Triglyceride levels; TC: Serum total cholesterol levels; TA-ALB: Time-averaged serum albumin; TA-SCr: Time-averaged serum creatinine; TA-BUN: Time-averaged blood urea nitrogen; TA-eGFR: Time-averaged eGFR; TA-UPE: Time-averaged 24-h urinary protein excretion; UA: Serum uric acid; UPE: 24-h urinary protein excretion; uRBC/HP: Hematuria; WBC: Blood leukocytes.

## Competing interests

The authors report no conflicts of interest. The authors alone are responsible for the content and writing of this paper.

## Authors’ contributions

We thank all of the doctors at the Nephrology Department of Ren Ji Hospital in Shanghai, China for their work. Research idea and study design: SM, ZN and YY; data acquisition: YY, QW, LC, XC, MZ, YX and CQ; data analysis: SM, ZN and YY; supervision or mentation :SM and ZN; manuscript writing: SM, ZN and YY. All authors read and approved the final manuscript.

## References

[B1] Tatematsu M Yasuda Y Morita Y Sakamoto I Kurata K Naruse T Yamamoto R Tsuboi N Sato W Imai E Matsuo S Maruyama S Complete remission within 2 years predicts a good prognosis after methylprednisolone pulse therapy in patients with IgA nephropathy Clin Exp Nephrol 2012 16 6 883 891 22618296 10.1007/s10157-012-0644-0

[B2] Le W Liang S Hu Y Deng K Bao H Zeng C Liu Z Long-term renal survival and related risk factors in patients with IgA nephropathy: results from a cohort of 1155 cases in a Chinese adult population Nephrol Dial Transplant 2012 27 4 1479 1485 21965586 10.1093/ndt/gfr527

[B3] Donadio JV Grande JP IgA nephropathy N Engl J Med 2002 347 10 738 748 12213946 10.1056/NEJMra020109

[B4] D’Amico G Influence of clinical and histological features on actuarial renal survival in adult patients with idiopathic IgA nephropathy, membranous nephropathy, and membranoproliferative glomerulonephritis: survey of the recent literature Am J Kidney Dis 1992 20 4 315 323 1415198 10.1016/s0272-6386(12)70293-7

[B5] Koyama A Igarashi M Kobayashi M Natural history and risk factors for immunoglobulin A nephropathy in Japan. Research Group on Progressive Renal Diseases Am J Kidney Dis 1997 29 4 526 532 9100040 10.1016/s0272-6386(97)90333-4

[B6] D’Amico G Natural history of idiopathic IgA nephropathy and factors predictive of disease outcome Semin Nephrol 2004 24 3 179 196 15156525 10.1016/j.semnephrol.2004.01.001

[B7] Disease K Improving Global Outcomes (KDIGO) Glomerulonephritis Work Group KDIGO Clinical Practice Guideline for Glomerulonephritis Kidney Int Suppl 2012 2 2 139 274

[B8] Berthoux F Mohey H Laurent B Mariat C Afiani A Thibaudin L Predicting the risk for dialysis or death in IgA nephropathy J Am Soc Nephrol 2011 22 4 752 761 21258035 10.1681/ASN.2010040355 PMC3065230

[B9] Xie J Kiryluk K Wang W Wang Z Guo S Shen P Ren H Pan X Chen X Zhang W Li X Shi H Li Y Gharavi AG Chen N Predicting progression of IgA nephropathy: new clinical progression risk score PLoS One 2012 7 6 e38904 22719981 10.1371/journal.pone.0038904 PMC3375310

[B10] Rauta V Gronhagen-Riska C IgA nephropathy: from predicting progression to treatment Duodecim 2006 122 2 215 222 16509071

[B11] Goto M Wakai K Kawamura T Ando M Endoh M Tomino Y A scoring system to predict renal outcome in IgA nephropathy: a nationwide 10-year prospective cohort study Nephrol Dial Transplant 2009 24 10 3068 3074 19515800 10.1093/ndt/gfp273 PMC2747499

[B12] Kaartinen K Syrjanen J Porsti I Hurme M Harmoinen A Pasternack A Huhtala H Mustonen J Inflammatory markers and the progression of IgA glomerulonephritis Nephrol Dial Transplant 2008 23 4 1285 1290 17986475 10.1093/ndt/gfm782

[B13] Wakai K Kawamura T Endoh M Kojima M Tomino Y Tamakoshi A Ohno Y Inaba Y Sakai H A scoring system to predict renal outcome in IgA nephropathy: from a nationwide prospective study Nephrol Dial Transplant 2006 21 10 2800 2808 16822793 10.1093/ndt/gfl342

[B14] D’Amico G Minetti L Ponticelli C Fellin G Ferrario F di Barbiano Belgioioso G Imbasciati E Ragni A Bertoli S Fogazzi G Prognostic indicators in idiopathic IgA mesangial nephropathy Q J Med 1986 59 363 378 3749442

[B15] Radford MG Jr Donadio JV Jr Bergstralh EJ Grande JP Predicting renal outcome in IgA nephropathy J Am Soc Nephrol 1997 8 2 199 207 9048338 10.1681/ASN.V82199

[B16] D’Amico G Natural history of idiopathic IgA nephropathy: role of clinical and histological prognostic factors Am J Kidney Dis 2000 36 2 227 237 10922300 10.1053/ajkd.2000.8966

[B17] Geddes CC Rauta V Gronhagen-Riska C Bartosik LP Jardine AG Ibels LS Pei Y Cattran DC A tricontinental view of IgA nephropathy Nephrol Dial Transplant 2003 18 8 1541 1548 12897092 10.1093/ndt/gfg207

[B18] Wakai K Kawamura T Matsuo S Hotta N Ohno Y Risk factors for IgA nephropathy: a case–control study in Japan Am J Kidney Dis 1999 33 4 738 745 10196018 10.1016/s0272-6386(99)70228-3

[B19] Levey AS Stevens LA Schmid CH Zhang YL Castro AF 3rd Feldman HI Kusek JW Eggers P Van Lente F Greene T Coresh J CKD-EPI (Chronic Kidney Disease Epidemiology Collaboration) A new equation to estimate glomerular filtration rate Ann Intern Med 2009 150 9 604 612 19414839 10.7326/0003-4819-150-9-200905050-00006 PMC2763564

[B20] Reich HN Troyanov S Scholey JW Cattran DC Remission of proteinuria improves prognosis in IgA nephropathy J Am Soc Nephrol 2007 18 12 3177 3183 17978307 10.1681/ASN.2007050526

[B21] Ballardie FW Roberts IS Controlled prospective trial of prednisolone and cytotoxics in progressive IgA nephropathy J Am Soc Nephrol 2002 13 1 142 148 11752031 10.1681/ASN.V131142

[B22] Lee HS Lee MS Lee SM Lee SY Lee ES Lee EY Park SY Han JS Kim S Lee JS Histological grading of IgA nephropathy predicting renal outcome: revisiting H.S. Lee’s glomerular grading system Nephrol Dial Transplant 2005 20 2 342 348 15618239 10.1093/ndt/gfh633

[B23] Yu HH Chiang BL Diagnosis and classification of IgA nephropathy Autoimmun Rev 2014 13 4–5 556 559 24434362 10.1016/j.autrev.2014.01.030

[B24] Delclaux C Jacquot C Callard P Kleinknecht D Acute reversible renal failure with macroscopic haematuria in IgA nephropathy Nephrol Dial Transplant 1993 8 3 195 199 8385283

[B25] Donadio JV Bergstralh EJ Grande JP Rademcher DM Proteinuria patterns and their association with subsequent end-stage renal disease in IgA nephropathy Nephrol Dial Transplant 2002 17 7 1197 1203 12105241 10.1093/ndt/17.7.1197

[B26] Bartosik LP Lajoie G Sugar L Cattran DC Predicting progression in IgA nephropathy Am J Kidney Dis 2001 38 4 728 735 11576875 10.1053/ajkd.2001.27689

[B27] Mackinnon B Fraser EP Cattran DC Fox JG Geddes CC Validation of the Toronto formula to predict progression in IgA nephropathy Nephron Clin Pract 2008 109 3 c148 c153 18663327 10.1159/000145458

[B28] Yasuda Y Horie A Odani H Iwase H Hiki Y Application of mass spectrometry to IgA nephropathy: structural and biological analyses of underglycosylated IgA1 molecules Contrib Nephrol 2004 141 170 188 14650232 10.1159/000074598

[B29] Vivante A Afek A Frenkel-Nir Y Tzur D Farfel A Golan E Chaiter Y Shohat T Skorecki K Calderon-Margalit R Persistent asymptomatic isolated microscopic hematuria in Israeli adolescents and young adults and risk for end-stage renal disease JAMA 2011 306 7 729 736 21846854 10.1001/jama.2011.1141

[B30] De Feo P Horber FF Haymond MW Meal stimulation of albumin synthesis: a significant contributor to whole body protein synthesis in humans Am J Physiol 1992 263 4 Pt 1 E794 E799 1415702 10.1152/ajpendo.1992.263.4.E794

[B31] Keane WF Zhang Z Lyle PA Cooper ME de Zeeuw D Grunfeld JP Lash JP McGill JB Mitch WE Remuzzi G Shahinfar S Snapinn SM Toto R Brenner BM RENAAL Study Investigators Risk scores for predicting outcomes in patients with type 2 diabetes and nephropathy: the RENAAL study Clin J Am Soc Nephrol 2006 1 4 761 767 17699284 10.2215/CJN.01381005

[B32] Tangri N Stevens LA Griffith J Tighiouart H Djurdjev O Naimark D Levin A Levey AS A predictive model for progression of chronic kidney disease to kidney failure JAMA 2011 305 15 1553 1559 21482743 10.1001/jama.2011.451

[B33] Staples AO Greenbaum LA Smith JM Gipson DS Filler G Warady BA Martz K Wong CS Association between clinical risk factors and progression of chronic kidney disease in children Clin J Am Soc Nephrol 2010 5 12 2172 2179 20813855 10.2215/CJN.07851109 PMC2994077

[B34] Yokoyama H Tomonaga O Hirayama M Ishii A Takeda M Babazono T Ujihara U Takahashi C Omori Y Predictors of the progression of diabetic nephropathy and the beneficial effect of angiotensin-converting enzyme inhibitors in NIDDM patients Diabetologia 1997 40 4 405 411 9112017 10.1007/s001250050694

[B35] Keane WF Brenner BM de Zeeuw D Grunfeld JP McGill J Mitch WE Ribeiro AB Shahinfar S Simpson RL Snapinn SM Toto R The risk of developing end-stage renal disease in patients with type 2 diabetes and nephropathy: the RENAAL study Kidney Int 2003 63 4 1499 1507 12631367 10.1046/j.1523-1755.2003.00885.x

[B36] Leehey DJ Kramer HJ Daoud TM Chatha MP Isreb MA Progression of kidney disease in type 2 diabetes - beyond blood pressure control: an observational study BMC Nephrol 2005 6 8 15985177 10.1186/1471-2369-6-8 PMC1180831

[B37] Kang SH Choi SR Park HS Lee JY Sun IO Hwang HS Chung BH Park CW Yang CW Kim YS Choi YJ Choi BS The Oxford classification as a predictor of prognosis in patients with IgA nephropathy Nephrol Dial Transplant 2012 27 1 252 258 21606384 10.1093/ndt/gfr295

[B38] Herzenberg AM Fogo AB Reich HN Troyanov S Bavbek N Massat AE Hunley TE Hladunewich MA Julian BA Fervenza FC Cattran DC Validation of the Oxford classification of IgA nephropathy Kidney Int 2011 80 3 310 317 21544062 10.1038/ki.2011.126

